# *Notes from the Field:* COVID-19 Vaccination Among Persons Living with Diagnosed HIV Infection — New York, October 2021

**DOI:** 10.15585/mmwr.mm7105a4

**Published:** 2022-02-04

**Authors:** James M. Tesoriero, Wendy Patterson, Demetre Daskalakis, Joyce Chicoine, Johanne Morne, Sarah Braunstein, Deepa T. Rajulu, Eli Rosenberg

**Affiliations:** ^1^New York State Department of Health AIDS Institute; ^2^Division of HIV Prevention, National Center for HIV, Viral Hepatitis, STD, and TB Prevention, CDC; ^3^New York City Department of Health and Mental Hygiene, New York, New York; ^4^New York State Department of Health Office of Public Health.

During March 1, 2020–October 26, 2021, approximately 2,500,000 COVID-19 cases and 58,000 COVID-19–associated deaths occurred in the state of New York.* New York has the highest U.S. per capita rate of persons living with diagnosed HIV infection (PLWDH),^†^ and population-level analyses adjusting for age, sex, and region have shown that PLWDH are more likely to be hospitalized for and to experience an in-hospital death from COVID-19 than are those not known to be PLWDH (*1*). CDC considers PLWDH who have a low CD4 cell count or who are not receiving HIV treatment to be at elevated risk for severe COVID-19–associated outcomes (*2*).

COVID-19 vaccines have been shown to be effective against symptomatic infection and hospitalization in New York, including during the period when the B.1.617.2 (Delta) and B.1.1.529 (Omicron) variants of SARS-CoV-2, the virus that causes COVID-19, predominated (*3*,*4*). PLWDH were an early priority group for vaccine eligibility, in part because of elevated COVID-19 risks. However, little is known about vaccination coverage among PLWDH.

Data from the New York State HIV surveillance registry were matched with the New York City Citywide Immunization Registry and New York State Immunization Information System. A deterministic matching algorithm^§^ was used to ascertain, as of October 24, 2021, COVID-19 vaccination status for PLWDH aged ≥18 years who were alive on December 31, 2020. Because death data were available only through December 2020, deaths in PLWDH reported in previous years in New York State, including 2020, were used to estimate that this analysis likely includes 500–1,000 PLWDH who died during the early part of 2021, precluding the opportunity for these decedents to begin or complete vaccination.

Persons were categorized as having received either a single dose of the Food and Drug Administration-authorized or -approved Ad.26.COV2.S (Janssen [Johnson & Johnson]) COVID-19 vaccine or 2 doses of the BNT162b2 (Pfizer BioNTech) or mRNA-1273 (Moderna) COVID-19 vaccines ≥14 days before October 24, 2021; only the first of a 2-dose series vaccine (Pfizer-BioNTech or Moderna); or having no matching vaccine record.^¶^ Booster and additional doses were not considered in this analysis. Consultation with the New York State Department of Health Institutional Review Board indicated that this work constitutes public health surveillance. This activity was reviewed by CDC and was conducted consistent with applicable federal law and CDC policy.**

Among 101,205 PLWDH included in the analysis,^††^ 64,278 (63.5%) had received either a single dose of Janssen vaccine or 2 doses of Pfizer-BioNTech or Moderna vaccine, 4,349 (4.3%) had received only 1 dose of Pfizer-BioNTech or Moderna vaccines, and 32,578 (32.2%) were unvaccinated (Table).

**TABLE:** Characteristics of persons living with diagnosed HIV infection in 2020 and COVID-19 vaccination status — New York, December 14, 2020–October 24, 2021

Characteristic	Study population, no.	COVID-19 vaccination status, no. (row %)*		
		Received 1 dose of Janssen vaccine or 2 doses of Pfizer-BioNTech or Moderna vaccine	Received only 1 dose of Pfizer-BioNTech or Moderna vaccine	Not vaccinated
**Total**	101,205	64,278 (63.5)	4,349 (4.3)	32,578 (32.2)
**Age group, yrs^†^**				
18–49	42,714	23,199 (54.3)	2,324 (5.4)	17,191 (40.2)
50–64	43,428	30,324 (69.8)	1,565 (3.6)	11,539 (26.6)
≥65	15,063	10,755 (71.4)	460 (3.1)	3,848 (25.5)
**Gender^†^**				
Men	70,636	45,788 (64.8)	2,836 (4.0)	22,012 (31.2)
Women	30,476	18,436 (60.5)	1,511 (5.0)	10,529 (34.5)
Nonconforming or nonbinary	93	54 (58.1)	2 (2.2)	37 (39.8)
**Race/Ethnicity**				
Black, non-Hispanic	45,534	26,691 (58.6)	2,413 (5.3)	16,430 (36.1)
White, non-Hispanic	23,208	16,420 (70.8)	677 (2.9)	6,111 (26.3)
Asian or Pacific Islander	2,519	1,728 (68.6)	54 (2.1)	737 (29.3)
Hispanic	29,075	18,906 (65.0)	1,172 (4.0)	8,997 (30.9)
Multiracial	590	359 (60.8)	24 (4.1)	207 (35.1)
American Indian or Alaska Native	190	111 (58.4)	7 (3.7)	72 (37.9)
Unknown	89	63 (70.8)	2 (2.2)	24 (27.0)
**Residence in 2020^§^**				
New York City	79,433	50,015 (63.0)	3,547 (4.5)	25,871 (32.6)
Rest of state of New York	21,772	14,263 (65.5)	802 (3.7)	6,707 (30.8)
**Virall**y **suppressed in 2020^¶^**				
No	25,307	9,650 (38.1)	1,326 (5.2)	14,331 (56.6)
Yes	75,898	54,628 (72.0)	3,023 (4.0)	18,247 (24.0)
HIV care in 2020**				
No	14,415	4,201 (29.1)	473 (3.3)	9,741 (67.6)
Yes	86,790	60,077 (69.2)	3,876 (4.5)	22,837 (26.3)

* Based on a Pearson chi-square test of statistical significance (p<0.001).

^†^ Current age and gender were determined as of March 31, 2021.

^§^ Residency was determined as last known residence in 2020.

^¶^ Viral suppression (<200 HIV RNA copies/mL) was determined at the last viral load test reported to the New York State HIV surveillance registry in 2020.

** HIV care was defined as any CD4, viral load, or genotype test reported to the New York State HIV surveillance registry in 2020.

Receipt of either a single dose of Janssen vaccine or 2 doses of Pfizer-BioNTech or Moderna vaccine increased with age, including 71.4% of PLWDH aged ≥65 years and 54.3% of those aged 18–49 years. Coverage was higher among men (64.8%) than women (60.5%), and among persons who identified as nonbinary or nonconforming (58.1%). Among racial/ethnic groups, coverage was highest among non-Hispanic White PLWDH (70.8%), and lowest among non-Hispanic Black (58.6%) and American Indian or Alaska Native persons (58.4%). Coverage was substantially lower among PLWDH who were not virally suppressed^§§^ at last test in 2020 (38.1%) compared with those who were (72.0%), and among those with no surveillance-based evidence of HIV care in 2020 (29.1%) than among those receiving care (69.2%).

This study found that COVID-19 vaccination coverage among PLWDH overall (63.5%) was lower than that in the general adult New York population (75.0%).^¶¶^ Differences in demographic composition between PLWDH and the general population might partly explain lower coverage; however, coverage was <75% across all examined PLWDH subgroups. Unmeasured factors, including socioeconomic status, might further explain the lower COVID-19 vaccination coverage among PLWDH.

Members of non-Hispanic Black and Hispanic communities are more likely to acquire SARS-CoV-2 and experience severe COVID-19–related outcomes than are those of other non-Hispanic and White communities (*5*,*6*). Gaps in vaccination coverage could thus serve to amplify disparities in COVID-19 outcomes among PLWDH. Addressing the large disparity in vaccination coverage by HIV care and viral suppression status is of particular importance, given the increased likelihood of severe COVID-19–related outcomes among PLWDH who experience immunocompromising conditions and considering the specific recommendation for additional doses for this group (*2*).

In addition to primary vaccination coverage, ensuring that PLWDH receive booster doses is critically important moving forward.*** Including COVID-19 vaccination in HIV-related service delivery might be effective at reducing disparities in vaccination coverage among PLWDH. For example, incorporating COVID-19 vaccination into existing HIV Data to Care^†††^ programming might help increase vaccination rates among PLWDH being relinked to HIV care. Similarly, leveraging HIV providers serving communities of color and sexual minority populations to promote COVID-19 vaccination could help to mitigate racial/ethnic and gender-based disparities in vaccination coverage.

## Footnotes

* https://coronavirus.health.ny.gov/covid-19-data-new-york (Accessed October 27, 2021).

† https://www.cdc.gov/hiv/library/reports/hiv-surveillance.html (Accessed January 23, 2022).

§ Data were matched using a deterministic matching algorithm implemented in SAS DataFlux (version 2.7; SAS Institute Inc.), which is used to link all routine and supplemental matches with the New York State HIV surveillance registry. https://www.sas.com/en_us/home. html (Accessed October 15, 2020).

¶ After a period of phased COVID-19 vaccine eligibility based on age, occupation, setting, or comorbidities beginning in December 2020, all New York residents aged ≥60 years were eligible for vaccination by March 10, 2021; eligibility was expanded to persons aged ≥30 years by March 30, and to all adults aged ≥18 years by April 6. HIV became a specific qualifying condition on February 15, 2021. https://www.governor.ny.gov/news/governor-cuomo-announces-new-yorkers-30-years-age-and-older-will-be-eligible-receive-covid-19

** 45 C.F.R. part 46.102(l)(2), 21 C.F.R. part 56; 42 U.S.C. Sect. 241(d); 5 U.S.C. Sect. 552a; 44 U.S.C. Sect. 3501 et seq.

†† Analyses excluded 4,163 PLWDH who were possible but not confirmed matches to the COVID vaccine registry. Analyses included 49 persons with a first vaccination date before December 1, 2020.

§§ Viral suppression is defined as <200 HIV RNA copies/mL at the last viral load test reported to the New York State HIV surveillance registry in 2020.

¶¶ https://coronavirus.health.ny.gov/covid-19-breakthrough-data (Accessed October 24, 2021).

*** https://www.cdc.gov/media/releases/2021/p1021-covid-booster.html (Accessed November 16, 2021).

††† https://wwwdev.cdc.gov/hiv/effective-interventions/treat/data-to-care/index.html?Sort=Title%3A%3Aasc&Intervention%20Name=Data%20to%20CareName=Data%20to%20Care (Accessed November 17, 2021).
